# Identification and Validation of a Three Pyroptosis-Related lncRNA Signature for Prognosis Prediction in Lung Adenocarcinoma

**DOI:** 10.3389/fgene.2022.838624

**Published:** 2022-07-19

**Authors:** Jichang Liu, Qiang Liu, Hongchang Shen, Yong Liu, Yadong Wang, Guanghui Wang, Jiajun Du

**Affiliations:** ^1^ Institute of Oncology, Shandong Provincial Hospital, Cheeloo College of Medicine, Shandong University, Jinan, China; ^2^ Department of Oncology, Shandong Provincial Hospital Affiliated to Shandong First Medical University, Jinan, China; ^3^ Department of Thoracic Surgery, Shandong Provincial Hospital, Cheeloo College of Medicine, Shandong University, Jinan, China

**Keywords:** lung adenocarcinoma, pyroptosis, prognosis, long noncoding RNA, immunotherapy

## Abstract

Pyroptosis, defined as programmed cell death, results in the release of inflammatory mediators. Recent studies have revealed that pyroptosis plays essential roles in antitumor immunity and immunotherapy efficacy. Long noncoding RNAs (lncRNAs) are involved in a variety of biological behaviors in tumor cells, although the roles and mechanisms of lncRNAs in pyroptosis are rarely studied. Our study aimed to establish a novel pyroptosis-related lncRNA signature as a forecasting tool for predicting prognosis and ascertaining immune value. Based on lung adenocarcinoma (LUAD) patients from The Cancer Genome Atlas (TCGA), we performed Pearson’s correlation analysis to identify pyroptosis-related lncRNAs. After differentially expressed gene analysis and univariate Cox regression analysis, we selected prognosis-related and differentially expressed lncRNAs. Finally, we performed multivariate Cox regression analysis to establish the three pyroptosis-related lncRNA signature. Kaplan–Meier (KM) survival analyses and receiver operating characteristic (ROC) curves indicated the excellent performance for predicting the prognosis of LUAD patients. At the same time, we applied multidimensional approaches to further explore the functional enrichment, tumor microenvironment (TME) landscape, and immunotherapy efficacy among the different risk groups. A nomogram was constructed by integrating risk scores and clinical characteristics, which was validated using calibrations and ROC curves. Three lncRNAs, namely, AC090559.1, AC034102.8, and AC026355.2, were involved in this signature and used to classify LUAD patients into low- and high-risk groups. Overall survival time (OS) was higher in the low-risk group than in the high-risk group, which was also validated in our LUAD cohort from Shandong Provincial Hospital. TME landscape analyses revealed that a higher abundance of infiltrating immune cells and a greater prevalence of immune-related events existed in the low-risk group. Meanwhile, higher expression of immune checkpoint (ICP) genes, higher immunophenoscore (IPSs), and greater T cell dysfunction in the low-risk group demonstrated a better response to immunotherapy than the high-risk group. Combined with predictions from the Tumor Immune Dysfunction and Exclusion (TIDE) website, we found that LUAD patients in the low-risk group significantly benefited from programmed cell death 1 (PD-1) and cytotoxic T-lymphocyte–associated protein 4 (CTLA4) immune checkpoint blockade (ICB) therapy compared with those in the high-risk group. Furthermore, drug susceptibility analysis identified potential sensitive chemotherapeutic drugs for each risk group. In this study, a novel three pyroptosis-related lncRNA signature was constructed, which could accurately predict the immunotherapy efficacy and prognosis in LUAD patients.

## Introduction

Lung cancer is currently the leading cause of cancer-related death worldwide, and its 5-year survival rate varies from 4% to 17% depending on stage and regional differences (Hirsch et al., 2017; Siegel et al., 2021). Lung adenocarcinoma (LUAD), as the most common subtype of lung cancer, accounts for approximately 40% of lung cancer cases (Denisenko et al., 2018). Despite the tremendous progress in a variety of treatment strategies, the survival rate of LUAD remains low. Therefore, there is an urgent need of developing accurate and reliable biomarkers for effective prognosis prediction of LUAD.

Pyroptosis is a novel programmed inflammatory cell death mediated by gasdermin proteins (GSDMs) (Yu P. et al., 2021; Deets and Vance, 2021). The activation of caspases-1/4/5/11 by inflammasomes mediates the cleavage of GSDMs, which results in the rupture of cell membranes and release of intracellular proinflammatory substances such as interleukin-1 β (IL-1 β) and interleukin-18 (IL-18). This is followed by a strong inflammatory response that is triggered in the immune microenvironment (Rathinam and Fitzgerald, 2016). In cancer, the complex effects of pyroptosis are dependent on genetic characteristics, which vary across different tissues. Several studies have reported that pyroptosis can suppress tumorigenesis, and even if only a few tumor cells undergo pyroptosis, a strong inflammatory response is triggered to recruit immune cells and enhance T cell-mediated antitumor immunity (Wang et al., 2020; Zhang et al., 2020). Another study has reported that pyroptosis also creates a tumor microenvironment (TME), which was a requirement of tumor growth (Zhang et al., 2021). Nevertheless, the mechanisms of action and effects of pyroptosis are still largely unknown in LUAD.

Long noncoding RNAs (lncRNAs) are transcripts longer than 200 nucleotides that are encoded by the genome but usually not translated into proteins (Bhan et al., 2017). Recent studies have reported that lncRNAs can regulate pyroptosis in a variety of ways such as targeting microRNAs (miRNA) and directly or indirectly binding to pyroptosis-associated proteins (Evavold, Hafner-Bratkovic et al., 2021). As lncRNAs can remain stable in the blood, which is easily collected from patients, they have good prospects as prognostic or predictive markers that are radiosensitive, chemosensitive, and sensitive to target therapy (Chen et al., 2021).

With the development of next-generation sequencing, various biomarkers have been identified to construct signatures for subgroup classification and prognosis prediction (Seijo et al., 2019; Lazzari et al., 2020). However, due to the lack of effective subgroup classification and prognostic prediction models, LUAD patients remain to be undertreated. Therefore, the construction of accurate subgroup classification and prognostic prediction models is urgently needed to guide clinicians on chemotherapy and immunotherapy.

In this study, we aimed to develop a prognostic risk model based on pyroptosis-related lncRNAs in LUAD patients. Based on LUAD patients from The Cancer Genome Atlas (TCGA), we constructed and validated a prognostic risk model to accurately predict the prognosis and overall survival (OS) of LUAD patients, which consisted of three pyroptosis-related lncRNAs. The risk score was significantly associated with tumor-infiltrating immune cells, immune function, and immunotherapy response. Our study revealed the potential connection and mechanism of action between pyroptosis, TME, and immunotherapy response.

## Materials and Methods

### Collection and Processing of Data Sets

Gene transcriptome profiling data, mutation data, and corresponding clinical information of LUAD patients were downloaded from (https://portal.gdc.cancer.gov/). Fragments per kilobase of exon model per million mapped fragments (FPKM) were converted to $log_{2}\;\left(FPKM+1\right)\;$ as a reflection of the gene expression level to visually display the results when constructing the figures. The dataset from TCGA served as a training cohort to construct the pyroptosis-related lncRNA prognostic model.

### Clinical LUAD Patient Specimens

A total of 45 LUAD patient specimens were recruited at Shandong Provincial Hospital, Shandong, China. The biomedical research ethic committee of Shandong Provincial Hospital approved this study (SWYX: NO. 2021-433).

### Differential Gene Expression and Mutation Analyses of Pyroptosis-Related Genes

We identified 33 pyroptosis-related genes from the literature, as listed in Supplementary Table S1 (Deets and Vance, 2021; Liu et al., 2021; Ye et al., 2021). The R software (R, vision 4.1.0) package “limma” (Ritchie et al., 2015) was used to determine the differently expressed pyroptosis-related genes with the absolute log2-fold change ( $\;|log_{2}\text{FC}|$ ) > 1 and adjusted *p* value <0.05. In addition, copy number variations (CNVs), mutation frequencies, and location analyses of pyroptosis-related genes were performed using “maftools” (Mayakonda et al., 2018) and “RCircos” with R software (Zhang et al., 2013).

### Identification of Pyroptosis-Related lncRNAs

Pyroptosis-related lncRNAs were extracted using Pearson’s correlation analysis between pyroptosis-related genes and lncRNAs (correlation coefficient >0.60, *p* < 0.001, and false-discovery rate (FDR) < 0.05).

### Construction of an mRNA–lncRNA Network

We extracted differently expressed pyroptosis-related lncRNAs with an $\left|log_{2}\mathrm{FC}\right|$ > 1 and an adjusted *p* value <0.05 using R package “limma”. Subsequently, 17 prognostic pyroptosis-related lncRNAs were identified by applying univariate Cox regression analysis (*p* < 0.05). A hazard ratio (HR) < 1 represented that lncRNAs were protective factors. To explore the regulatory mechanism of the selected pyroptosis-related lncRNAs, an mRNA–lncRNA regulatory network was constructed based on the 17 lncRNAs and two co-expressed mRNAs.

### Survival Analysis and Differential Expression Analysis of Co-expressed mRNAs by the Website

The Kaplan–Meier plotter (https://kmplot.com/analysis/) was used to analyze the impact of co-expressed mRNAs, while TIMER2.0 (Li et al., 2020) (http://timer.cistrome.org/) and gene expression profiling integrative analysis (Tang et al., 2017) (GEPIA, http://gepia.cancer-pku.cn/) were used for differential expression analysis of co-expressed mRNAs.

### Construction of a Risk Score

According to the prognostic-related lncRNAs, multivariate Cox regression analysis was performed to identify the best prognostic signature, which consisted of three lncRNAs, namely, AC090559.1, AC026355.2, and AC034102.8. We calculated the risk score as follows: risk score = (−0.2603×AC090559.1) + (−0.0974×AC026355.2) + (−0.9235×AC034102.8). Based on the median of risk score, the LUAD patients from the TCGA database were divided into high- and low-risk groups for further analysis. Subsequently, risk score distribution maps, survival status maps, and lncRNA expression heat maps were plotted. Kaplan–Meier analysis was applied to compare the overall survival (OS) of the two groups. The time receiver operating characteristic (ROC) curve was plotted using the “timeROC” package with R software, which was used to evaluate the predictive capability of the risk model. The area under the curve (AUC) of the constructed risk model in predicting the OS of LUAD patients was compared with several previously published lncRNA signatures, including the ferroptosis-related lncRNA signature of Lu (Lu et al., 2021), hypoxia-associated lncRNA signature of Shao (Shao et al., 2021), autophagy-related lncRNA signature of Liu (Liu and Yang, 2021), and six-lncRNA-based prognostic signatures of Yang (Yang et al., 2021).

### Establishment of the Nomogram

The prognostic significance of the risk score and other clinical characteristics was evaluated by using univariate and multivariate Cox regression analyses. A nomogram was established to predict the 1-, 3-, and 5-year OS, which consisted of the risk score, age, and stage. Calibration curves were plotted to assess the accuracy of the risk model.

### Functional Enrichment Analysis

GO enrichment analysis was applied to investigate the potential pathways of the differentially expressed pyroptosis genes using “org.Hs.eg.db,” “clusterProfiler,” and “enrichplot” modules within the R package. By applying the “GSVA” tool, GSVA enrichment analysis was performed based on hallmark gene sets extracted from the MSigDB database (Hänzelmann et al., 2013).

### TME Landscape Analyses

Single-sample gene set enrichment analysis (ssGSEA) was performed to compare the difference in abundance of 28 types of infiltrating immune cells, 13 immune functions, and 13 other tumor-related biological processes, which were extracted from previous articles (Şenbabaoğlu et al., 2016; Charoentong et al., 2017; Mariathasan et al., 2018). In addition, we used computational methods to assess the infiltrating immune cells, including the TIMER (Li et al., 2020), CIBERSORT (Chen et al., 2018), quanTIseq (Finotello et al., 2019), MCP-counter (Becht et al., 2016), xCell (Aran et al., 2017), EPIC (Racle et al., 2017), and TIDE (Jiang et al., 2018) algorithms. Immune score and tumor purity were calculated using the “ESTIMATE” tool within the R package (Chakraborty and Hossain, 2018). In addition, correlation analysis was performed using the correlation heat map tool in HiPlot (https://hiplot.com.cn), a comprehensive web platform for scientific data visualization.

### Prediction of Response to Immune Checkpoint Blockade (ICB) Therapy

The immunophenoscore (IPS) was obtained from The Cancer Immunome Atlas (https://tcia.at/). Information on the dysfunction and exclusion of infiltrating cytotoxic T lymphocytes (CTLs) was downloaded from the Tumor Immune Dysfunction and Exclusion (TIDE) website (http://tide.dfci.harvard.edu/). In addition, TIDE was used to evaluate patients who received a benefit or no benefit from ICB therapy through the comprehensive biomarkers of the ICB response in different groups (Fu et al., 2020).

### RNA Extraction and Real-Time PCR

Following the manufacturer’s protocol, total RNA was extracted from clinical specimens using AG RNAex Pro Reagent (Accurate Biotechnology (Hunan) Co., Ltd., China). The Evo M-MLVRT Master Mix kit (Accurate Biotechnology (Hunan) Co., Ltd., China) was used for reverse transcription to obtain cDNA. Relative gene expression was detected using the SYBR Premix Ex Tap kit (Accurate Biotechnology (Hunan) Co., Ltd., China) and normalized to the expression using 18S. The primers are listed in Supplementary Table S1.

### Statistical Analysis

Student’s t test was used to compare the differences between the two groups. Pearson’s correlation test was used for correlation analysis. Survival analysis was performed by the Kaplan–Meier method and compared with the log-rank test. All statistical analyses were performed using R software (vision 4.1.0), and *p* < 0.05 was considered statistically significant.

## Results

### Landscape of Pyroptosis-Related Genes in LUAD

**FIGURE 1:**
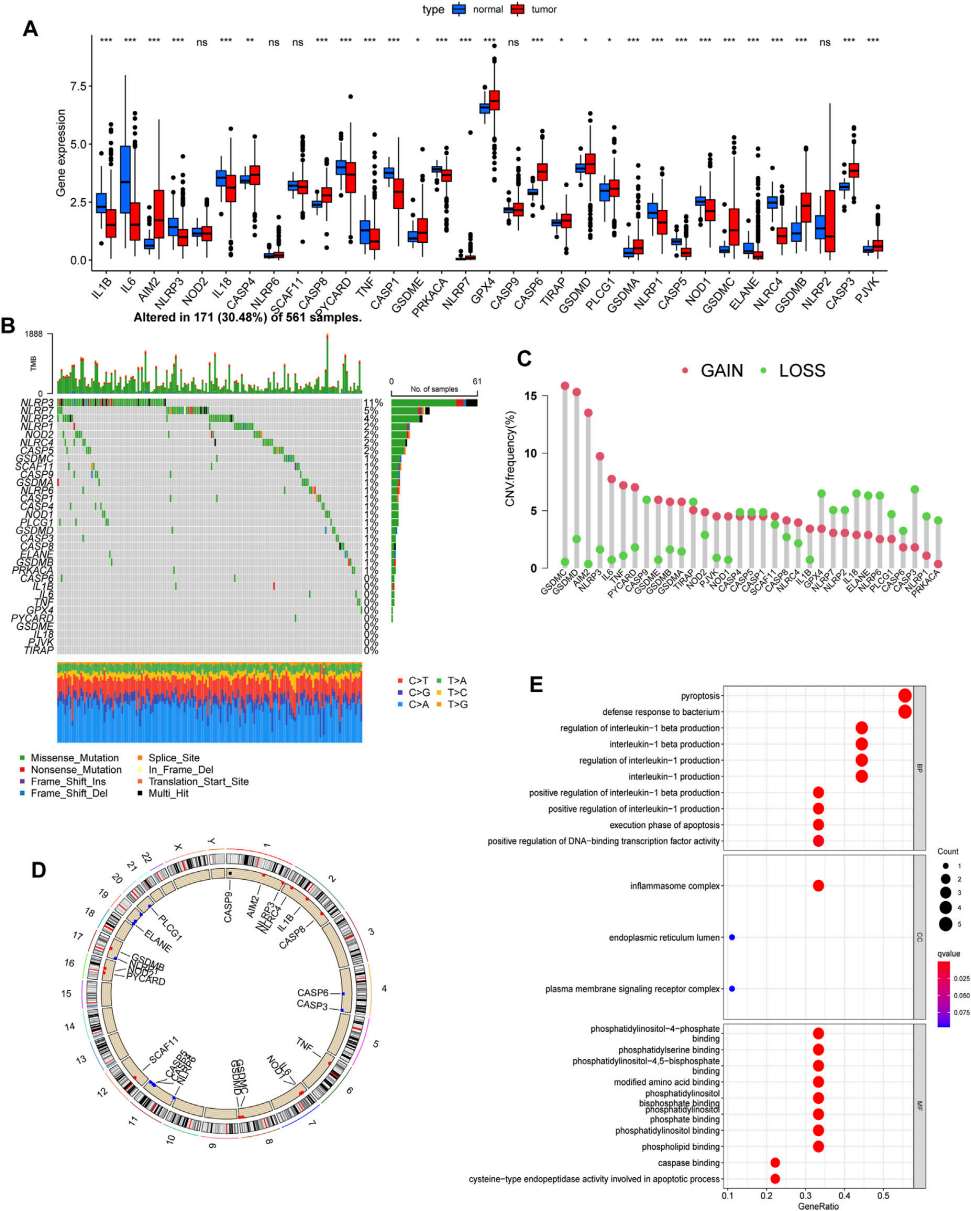
Landscape of expression, genetic variation, and functional enrichment of pyroptosis-related genes in LUAD. **(A)** Expression of pyroptosis-related genes between normal tissues and LUAD tissues (Wilcoxon test, *p < 0.05; **p < 0.01; ***p < 0.001; ns, not statistically significant). **(B)** Landscape of mutation profiles in LUAD patients from the TCGA cohort. **(C)** CNV frequency of 33 pyroptosis-related genes. **(D)** Location of CNV alternation of pyroptosis-related genes in the chromosome. The red dots represent more samples with increased copy number gains than samples with copy number losses, while the blue dots are the opposite. The black dot means the two are equal. **(E)** Enrichment analysis of GO biological process, cellular component, and molecular function. LUAD, lung adenocarcinoma; CNV, copy number variation.

We identified 33 pyroptosis-related genes and performed differential gene expression analysis between LUAD and normal lung tissues. A total of 15 genes, namely, AIM2, CASP4, CASP8, GSDME, NLRP7, GPX4, CASP6, TIRAP, GSDMD, PLCG1, GSDMA, GSDMC, GSDMB, CASP3, and PJVK were highly expressed in LUAD, while the expression of IL1B, IL6, NLRP3, IL18, PYCARD, TNF, CASP1, PRKACA, NLRP1, CASP5, NOD1, ELANE, and NLRC4 was decreased (Figure 1A). Subsequently, we developed a panorama of the somatic mutations of pyroptosis-related genes. A total of 30.48% of the samples had mutations, and the three genes with the highest mutation rates were NLRP3 (11%), NLRP7 (5%), and NLRP2 (4%) (Figure 1B). Figures 1C and D show the frequency of the CNV alterations of the 33 pyroptosis-related genes and their locations on the chromosome. The frequency of copy number mutations in pyroptosis-related genes was greater than the frequency of copy number deletions. To clarify the functions of the pyroptosis-related genes, we conducted GO functional enrichment analyses. Apart from the pyroptosis pathway, pyroptosis-related genes were also enriched in defense response to bacterium, regulation of interleukin-1 or interleukin-1β production, and inflammasome complex (Figure 1E).

### Identification of Prognostic Pyroptosis–Related lncRNAs

**FIGURE 2:**
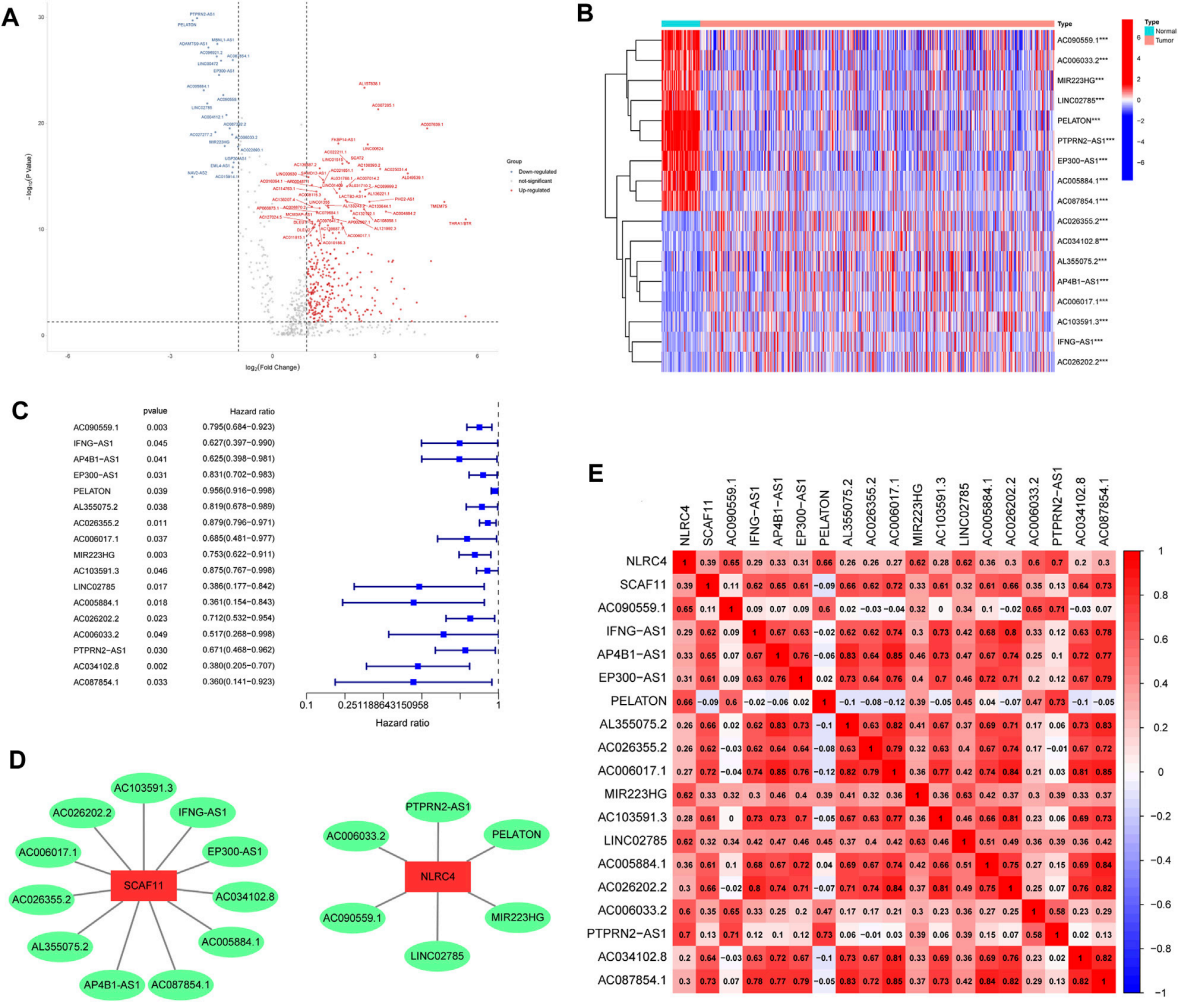
Identification and characteristics of pyroptosis-related lncRNAs. **(A)** Volcano plot showing the differently expressed pyroptosis-related lncRNAs. **(B)** Heat map visualizes the differential expression of prognostic pyroptosis-related lncRNAs between normal and LUAD. **(C)** Forest plot showing the result of univariate Cox regression analysis for screening prognosis-related lncRNAs (*p* < 0.05). **(D)** Interaction network of the 17 prognostic prognosis-related lncRNAs–mRNAs. **(E)** Visualization of prognosis-related lncRNA–mRNA correlation. ***p < 0.001, LUAD, lung adenocarcinoma.

We first performed correlation analysis (correlation coefficients >0.60, *p* < 0.001, FDR<0.05) between 14,057 lncRNAs and 33 pyroptosis-related genes in LUAD samples, and 1,070 pyroptosis-related lncRNAs were identified. Subsequently, 320 differently expressed pyroptosis-related lncRNAs were identified and exhibited in a volcano map after differential expression analysis (Figure 2A). Finally, 17 lncRNAs related to LUAD prognosis were identified after applying univariate COX regression analysis (Table 1). A heat map was plotted to show the differential expression of the 17 lncRNAs between normal and tumor tissues (Figure 2B). The forest map indicated that the hazard ratios of these 17 lncRNAs were all <1, suggesting that they were protective factors for prognosis (Figure 2C). In addition, we used Cytoscope to construct a co-expression network for the 17 pyroptosis-related lncRNAs and two corresponding genes (Figure 2D). The correlation scores between NLRC4, SCAF11, and 17 lncRNAs are shown in Figure 2E, suggesting that these lncRNAs may perform functions through NLRC4 and SCAF11. Next, we analyzed the impact of NLRC4 and SCAF11 on survival by applying the Kaplan–Meier plotter, which indicated that high expression was significantly associated with high overall survival (Supplementary Figure S1A). TIMER2.0 and GEPIA were used for differential gene expression analysis, and the results showed a significant downregulation of NLRC4 in LUAD but no change in SCAF11 (Supplementary Figure S1B–D).

**TABLE 1:** Results of univariate Cox regression.

ID	HR	HR.95L	HR.95H	*p*-Value
AC090559.1	0.79477	0.68402	0.923452	0.002699
IFNG-AS1	0.626928	0.396886	0.990309	0.045315
AP4B1-AS1	0.624799	0.397789	0.98136	0.041187
EP300-AS1	0.831072	0.702447	0.983248	0.031015
PELATON	0.955892	0.915806	0.997732	0.039035
AL355075.2	0.818529	0.677601	0.988767	0.037789
AC026355.2	0.879216	0.79625	0.970827	0.010915
AC006017.1	0.685354	0.48061	0.97732	0.036918
MIR223HG	0.752638	0.622047	0.910645	0.003471
AC103591.3	0.874798	0.767129	0.997579	0.045919
LINC02785	0.386337	0.17719	0.842352	0.016787
AC005884.1	0.360554	0.154291	0.842556	0.018495
AC026202.2	0.712373	0.532036	0.953836	0.022767
AC006033.2	0.517374	0.268118	0.99835	0.049427
PTPRN2-AS1	0.671347	0.4684	0.962225	0.030035
AC034102.8	0.380262	0.204576	0.706823	0.002236
AC087854.1	0.360318	0.140624	0.923239	0.033476

### Establishment of a Risk Model

**FIGURE 3:**
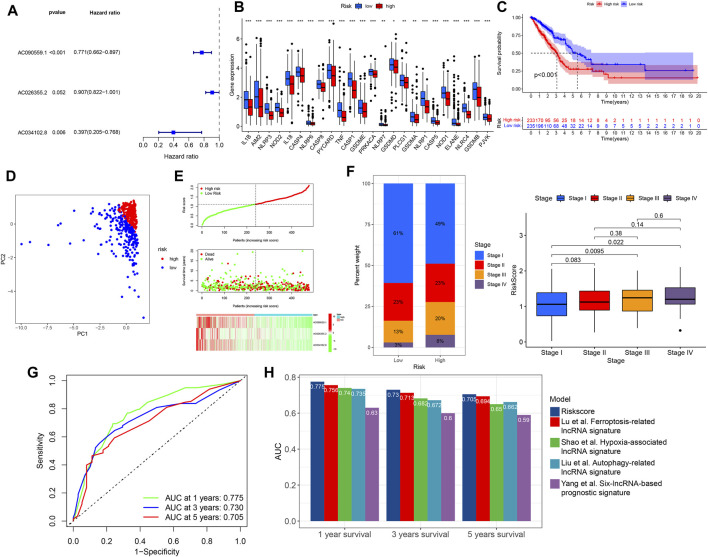
Construction of risk model and clinical correlation of high- and low-risk groups. **(A)** Three lncRNAs were identified in multivariate Cox regression analysis for model construction. **(B)** Differential expression of pyroptosis-related genes between high- and low-risk groups (Wilcoxon test, *p < 0.05; **p < 0.01; ***p < 0.001). **(C)** Kaplan–Meier curve of high- and low-risk groups. **(D)** PCA of high and low-risk groups. **(E)** Risk curve based on the risk score of each sample. Scatterplot showing the survival status of LUAD patients. Heat map showing the expression of identified lncRNAs in high- and low-risk groups. **(F)** Relationship between tumor stage and risk score. **(G)** Time-dependent ROC curves of OS at 1, 3, and 5 years. **(H)** Comparison of the risk model with four published lncRNA signatures. OS, overall survival; PCA, principal component analysis.

We subsequently performed multivariate COX regression analysis on the previously obtained 17 lncRNAs and three lncRNAs were identified (Figure 3A). A risk score was established based on the multivariate regression coefficients (Table 2). According to the median risk score, LUAD patients were divided into high- and low-risk groups. Most pyroptosis-related genes were significantly upregulated in the low-risk group, suggesting a more active involvement of pyroptosis (Figure 3B). By contrast, survival analysis revealed that patients in the low-risk group showed a better prognosis, indicating that pyroptosis was associated with survival advantages (Figure 3C). Principal component analysis (PCA) indicated significant distinction in transcription profiles between the two groups (Figure 3D). The risk score, survival status, and lncRNA expression are shown in Figure 3E, revealing that mortality was significantly related to risk score. Moreover, a higher risk score was significantly related to advanced stage (Figure 3F). These results indicated that a lower risk score was associated with active pyroptosis and better clinical outcome. The areas under the curves (AUCs) of the 1-year, 3-year, and 5-year ROC curve were 0.775, 0.730, and 0.705, respectively, revealing a high accuracy in the prognosis prediction of the risk model (Figure 3G). Compared with four published lncRNA signatures, the risk model we constructed had higher accuracy in predicting 1-, 3-, and 5-year survival for TCGA–LUAD patients (Figure 3H).

**TABLE 2:** Results of multivariate Cox regression.

ID	Coef	HR	HR.95L	HR.95H	*p*-Value
AC090559.1	−0.26027	0.77084	0.662457	0.896955	0.000761
AC026355.2	−0.09742	0.907175	0.822133	1.001013	0.052403
AC034102.8	−0.9235	0.397127	0.205362	0.76796	0.006058

### Construction of a Predictive Nomogram

**FIGURE 4:**
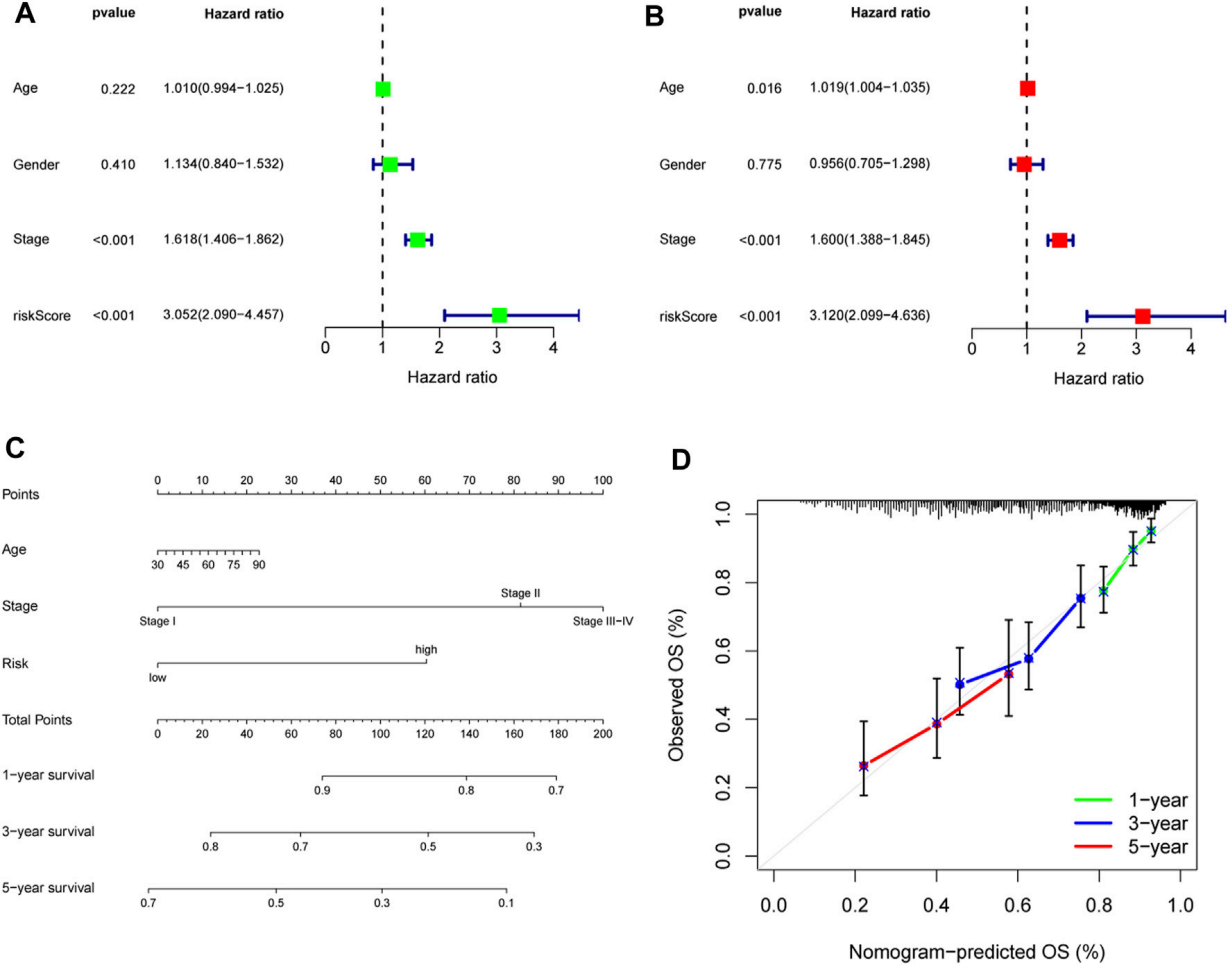
Construction and validation of a nomogram. Univariate **(A)** and multivariate **(B)** Cox regression analyses of age, gender, stage, and risk score. **(C)** Nomogram for predicting the OS of LUAD patients at 1, 3, and 5 years. **(D)** Calibration curves of the nomogram for OS prediction at 1, 3, and 5 years. OS, overall survival; LUAD, lung adenocarcinoma.

After incorporating the risk scores and clinical features, univariate and multivariate COX regression analyses were performed, and the results indicated that the risk score could serve as an independent factor affecting the survival of LUAD patients, similar to stage and age (Figures 4A,B). To better predict the 1-, 3-, and 5-year OS of LUAD patients, we constructed a nomogram by incorporating risk score, age, and stage (Figure 4C), and calibration curves were constructed to assess the accuracy of nomogram (Figure 4D).

### Differences in Landscape of the TME Between the High- and Low-Risk Groups

**FIGURE 5:**
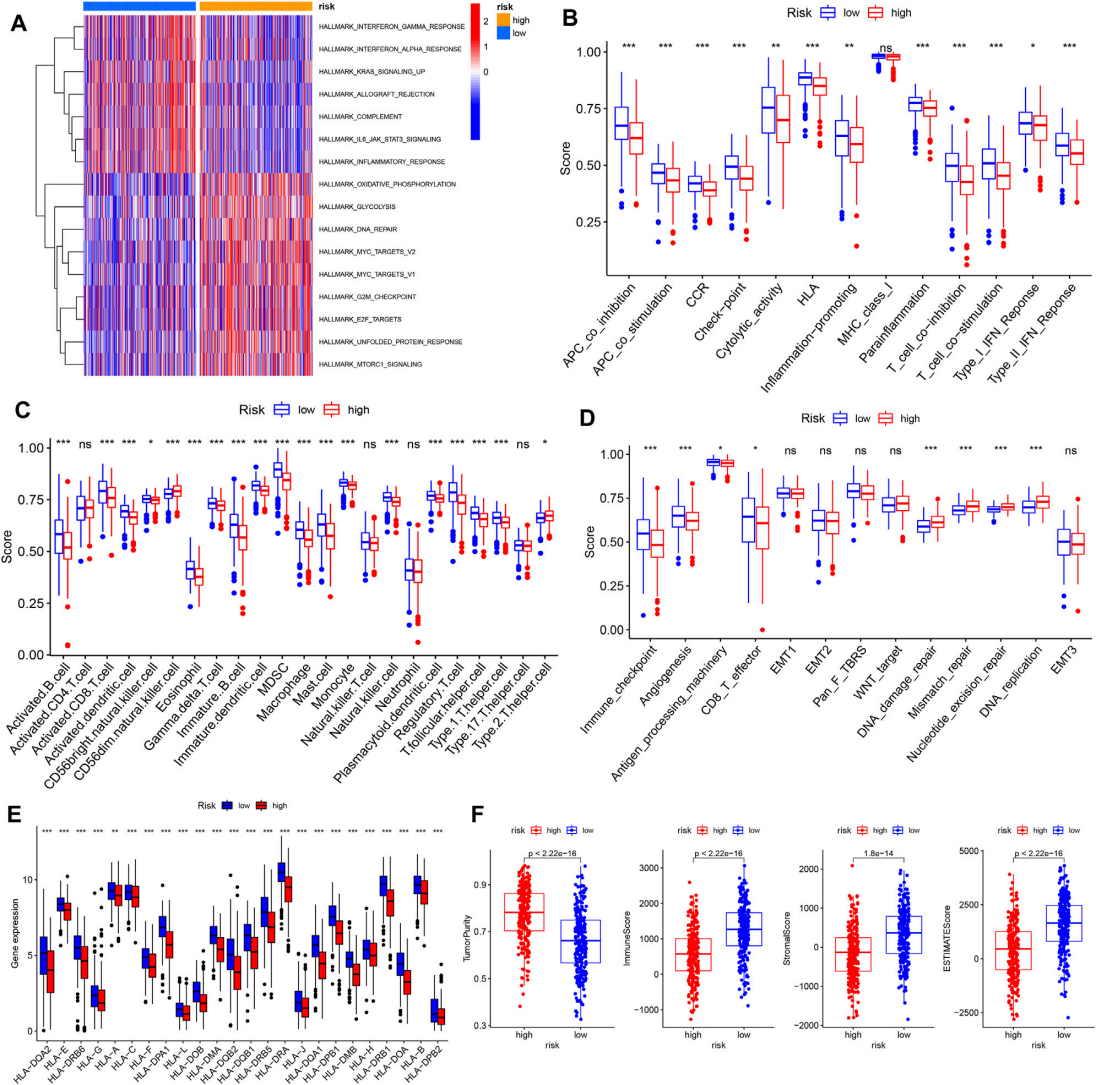
Differences in landscape of the TME between high- and low-risk groups. **(A)** GSVA enrichment analysis of tumor hallmark pathways. **(B–E)** Differences in immune-related functions, TME infiltrating immune cells, other tumor-related functions, and HLA-related gene expression in the high- and low-risk groups. **(F)** Comparison of tumor purity, immune score, stromal score, and ESTIMATE score between high- and low-risk groups. TME, tumor microenvironment, *p < 0.05; **p < 0.01; ***p < 0.001; ns, not statistically significant.

To further understand the significance of the risk score, we conducted GSVA analysis. Several immune-related pathways and events, including interferon (IFN) gamma/alpha response, IL-6–JAK-STAT3 signaling, allograft rejection, and inflammatory response, were upregulated in the low-risk group, while in the high-risk group, metabolic and cancer-promoting pathways, such as oxidative phosphorylation, glycolysis, MYC signaling, E2F signaling, and MTORC1 signaling, were activated (Figure 5A).

We investigated the differences in various immune-related functions, immune-infiltrating cells, and other tumor-related functions between high- and low-risk groups using ssGSEA. As expected, the low-risk group, with a higher level of pyroptosis, was more involved in immune-related functions such as antigen-presenting cell (APC) co-stimulation/inhibition, inflammation, cytolytic activity, human leukocyte antigen (HLA) function, T cell co-stimulation/inhibition, and type I/II IFN responses, indicative of active immune functions (Figure 5B). Consistently, the higher abundance of most infiltrating immune cells existed in the low-risk group, including activated B cells, activated CD8^+^ T cells, dendritic cells, eosinophils, MDSCs, and macrophages (Figure 5C, Supplementary Figure S2). In addition, several tumor-related pathways and events, including immune checkpoint, angiogenesis, antigen processing machinery, and CD8^+^ T effector function, were also upregulated in the low-risk group. By contrast, DNA damage repair, mismatch repair, nucleotide excision repair, and DNA replication were upregulated in the high-risk group, which may play roles in genome stability and LUAD progression (Figure 5D). In addition, HLA-related genes were significantly upregulated in the low-risk group, indicative of antigen presentation (Figure 5E). Finally, tumor purity, immune score, ESTIMATE score, and stromal score were calculated using the “estimate” tool within the R package (Figure 5F). Significantly upregulated immune and stromal scores were features of the low-risk group, while the high-risk group was characterized by higher tumor purity.

### Prediction of Response to ICB Therapy

**FIGURE 6:**
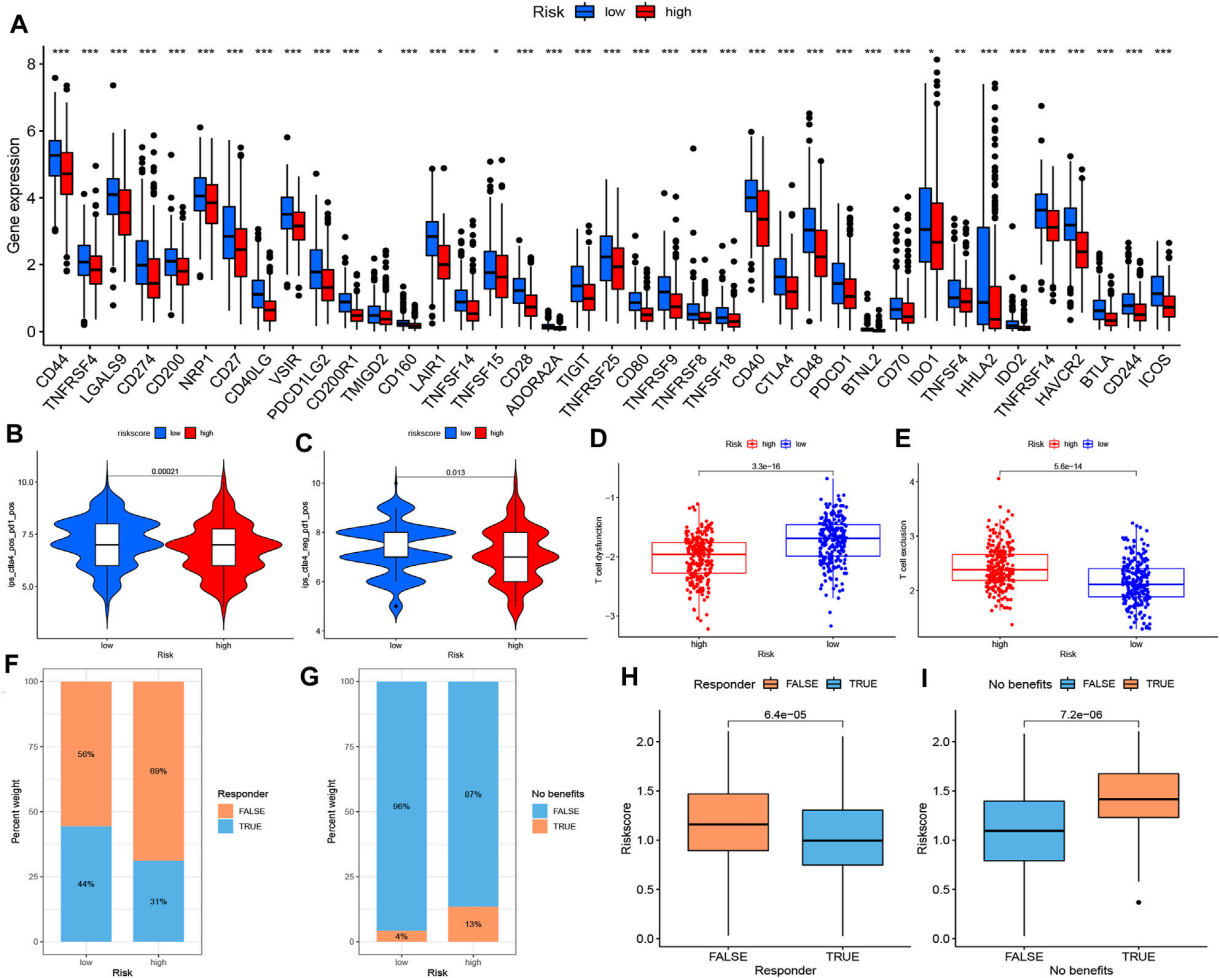
Prediction of response to ICB therapy. **(A)** The expression of ICPs between high- and low-risk groups (Wilcox test, *p < 0.05; **p < 0.01; ***p < 0.001). The response to combined PD1 and CTLA4 blockade therapy **(B)** and PD1 monotherapy **(C)** between high- and low- risk groups. Differences in T cell status, including T cell dysfunction **(D)** and T cell exclusion **(E)**, in high and low risk groups. **(F–I)** Prediction of the “responder” and “no benefit” of PD1 and CTLA4 blockade therapy in TCGA- LUAD patients from TIDE website. ICB immune checkpoint blockade, ICP immune checkpoint, PD1 programmed cell death protein 1, TCGA The Cancer Genome Atlas, LUAD lung adenocarcinoma.

Considering the significant differences in the TME landscape, we identified several ICPs and performed differential expression analysis between low- and high-risk groups. As shown in Figure 6A, all 39 selected ICPs were upregulated in the low-risk group, indicating the potential benefit of ICI therapy. Furthermore, we used IPS obtained from TIDE as a predictor of the response to anticytotoxic T lymphocyte antigen-4 (CTLA-4) and antiprogrammed cell death protein 1 (anti-PD-1) antibodies (Charoentong et al., 2017). The results showed a higher IPS level in the low-risk group, revealing a better response to combined PD1 and CTLA4 blockade therapy or PD1 monotherapy (Figures 6B,C). Considering the upregulation of immune checkpoints and infiltration of Treg cells in the low-risk group, which could suppress the effect of CD8^+^ T cells, we further investigated the status of T cells in the TME. Consistently, the low-risk group was characterized by higher T cell dysfunction and lower exclusion, suggesting a potential advantage to ICI therapy (Figures 6D,E). To further investigate the response to ICIs, we utilized the TIDE website to predict the “responder” and “nonresponders” of PD1 and CTLA4 blockade therapy in TCGA–LUAD patients, which were constructed by integrating TIDE score, IFNG, MSI, MDSC, CAFs, and other published modules. We found that 44% of patients in the low-risk group were identified as responders to PD1 and CTLA4 blockade therapy, while only 31% in the high-risk group were classified as responders (Figure 6F). In further analysis, only 4% of patients in the low-risk group showed no benefit from ICB compared with 13% in the high-risk group (Figure 6G). In addition, patients who were not responders or showed no benefit from PD1 and CTLA4 blockade therapy had significantly higher risk scores (Figures 6H,I).

In conclusion, the low-risk group could be clarified as an immune “hot” phenotype, with a high abundance of infiltrating immune cells and better efficacy for PD1 and CTLA4 blockade therapy, while the high-risk group represented the immune “cold” phenotype which was characterized by less sensitivity to ICB.

To explore whether the risk score affected immune function through NLRC4 or SCAF11, we performed a correlation analysis between NLRC4 or SCAF11 and infiltrating immune cells. The heat map showed that lncRNA AC090559.1 and its corresponding mRNA NLRC4 were closely correlated with infiltrating immune cells, suggesting that AC090559.1 may exert its regulatory function by targeting NLRC4 (Supplementary Figure S3). The scatter plots of NLRC4, SCAF11, and various infiltrating immune cells downloaded from TIMER indicated that NLRC4 was highly correlated with a variety of immune cells (Supplementary Figure S4). Finally, we predicted the sensitivity of several common chemotherapy drugs in high- and low-risk groups. The high-risk group was more sensitive to doxorubicin, sorafenib, docetaxel, and erlotinib but less sensitive to gefitinib (Supplementary Figure S5).

### Validation of the Risk Model With the Clinical Cohort

**FIGURE 7:**
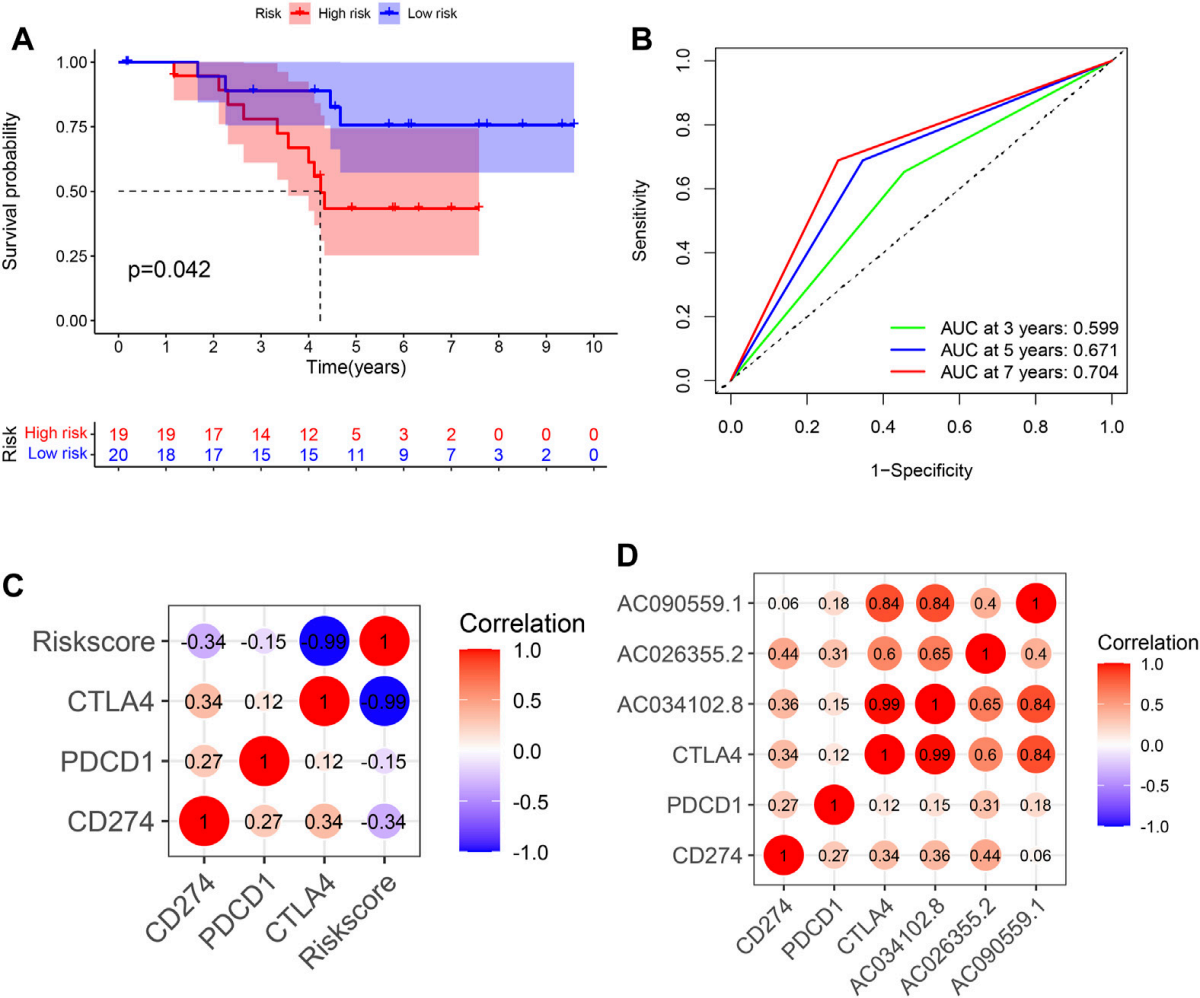
Validation of risk score using clinical specimens of LUAD patients. **(A)** Kaplan–Meier curve of high- and low-risk groups in clinical LUAD patients. **(B)** Time-dependent ROC curves of OS at 1, 3, and 5 years. **(C)** The correlation between 3 identified lncRNAs, risk score and immune checkpoints. LUAD, lung adenocarcinoma; OS, overall survival.

We detected the relative expression levels of the three lncRNAs in LUAD specimens by using qRT-PCR. The risk score was subsequently calculated, and the patients were divided into high- and low-risk groups based on the median value. The overall survival of the low-risk group was significantly better, consistent with previous results (Figure 7A). The AUC values of 3 year, 5 year and 7 year were 0.599, 0.671, and 0.704, respectively (Figure 7B). By correlation analysis of the three identified lncRNAs and immune checkpoint genes in LUAD specimens, we found a strong correlation between risk score and CTLA4, indicative of a better response to anti-CTLA4 immunotherapy (Figures 7C,D).

## Discussion

In recent years, the development of immunotherapy and chemotherapy has brought about a paradigm shift in the treatment of LUAD patients. However, only some patients can benefit from immunotherapy due to drug resistance (Boumahdi and de Sauvage, 2020). Furthermore, it is still difficult to identify patients who may benefit from immunotherapy. Therefore, it is important to identify novel therapeutic targets.

Pyroptosis is a type of GSDM-mediated programmed cell death that is accompanied by the release of damaged-associated molecular pattern (DAMP) and mature IL-1 proteins, which can lead to recruitment of immune cells (Rathinam and Fitzgerald, 2016; Liu et al., 2021). Pyroptosis plays distinct roles in tumor microenvironments, and thus, tumorigenesis. On the one hand, long-term chronic inflammation induced by pyroptosis can stimulate and promote tumorigenesis. On the other hand, pyroptosis can also alter the abundance of infiltrating immune cells and transform “cold” tumors into “hot” tumors, which could enhance the antitumor immune response (Du et al., 2021; Zhang et al., 2021). Moreover, pyroptosis plays important roles in activating antitumor immunity. Granzyme A, which is released by CTLs and natural killer cells, can directly cleave GSDMB to mediate tumor cell pyroptosis (Zhou et al., 2020). Meanwhile, the inflammatory response triggered by pyroptosis in a small proportion of tumor cells can trigger a strong antitumor immune response (Wang et al., 2020). Similarly, GSDME also converts apoptosis, a noninflammatory programmed cell death, into pyroptosis, and exerts antitumor functions while enhancing CTL and natural killer cell infiltration (Zhang et al., 2020). Therefore, we speculate that pyroptosis can improve the prognosis and efficacy of immunotherapy in LUAD patients. In addition, abnormally expressed lncRNAs in tumors can be detected in plasma, urine, or saliva specimens, which indicates that they have potential value as biomarkers of lncRNAs (Chandra Gupta and Nandan Tripathi, 2017). Therefore, we aimed to develop a pyroptosis-related lncRNA-based signature to accurately predict the immunotherapy efficacy and prognosis of LUAD patients.

In this study, we first extracted pyroptosis-related genes from previously published articles, and 17 prognostic-related lncRNAs were identified after correlation analysis, differential expression analysis, and univariate Cox regression analysis. After multivariate regression analysis, we developed a risk model consisting of three pyroptosis-related lncRNAs. Based on the median risk score, we divided patients into high- and low-risk groups and analyzed the differences in prognosis and immune microenvironment. ROC curves were used to verify the accuracy of the risk score. In addition, 39 LUAD patients from Shandong Provincial Hospital were used as a test cohort to verify the risk score.

The risk score served as an independent factor, which was significantly associated with stage and survival. A better prognosis was observed in the low-risk group, accompanied by highly expressed pyroptosis-related genes. Consistent with previous studies, active pyroptosis was associated with better prognosis. GSVA enrichment analysis showed that a greater inflammatory response existed in the low-risk group, which involved interferon γ/α and IL6/JAK/STAT3 pathways, allograft rejection, complement, and inflammatory response. By contrast, the high-risk group was more related to MYC, E2F, MTORC1, and glycolytic pathways, which are essential for tumorigenesis. Similarly, the abundance of infiltrating immune cells was generally higher in the low-risk group. Considering the results of ssGSEA (Figure 5C) and correlation analysis (Supplementary Figure S3), we noticed that the significantly enriched infiltrating immune cells were mainly associated with inflammation and antigen presentation (eosinophils, mast cells, monocytes, macrophages, immature dendritic cells, and activated dendritic cells) and immunomodulatory events (regulatory T cell and various T helper cells) in the low-risk group. As shown in Figure 5E, HLA function was significantly activated in the low-risk group, further confirming antigen presentation.

Interestingly, activated CD8^+^ T cells highly infiltrated the low-risk group, accompanied by the high abundance of immunosuppressive cells such as Treg and MDSCs (Figure 5C). Considering simultaneous T cell co-stimulation and co-inhibition, we examined whether T cells could exercise their conventional function of antitumor immunity in the low-risk group (Figure 5B). As such, we analyzed the dysfunction and exclusion of T cells, which revealed significant T cell dysfunction in the low-risk group (Figures 6D,E). A previous study has reported that a broad spectrum of dysfunctional states can exist in intratumoral T cells, which essentially blocks the durable clinical benefits of patients (Thommen and Schumacher, 2018). The upregulation of ICPs mediated by PD-1 was the main manifestation of T cell dysfunction, accompanied by a variety of other inhibitory factors such as Treg cell infiltration, cytokine production, and metabolic stress (Speiser et al., 2016; Zarour, 2016; Gajewski et al., 2013). According to previous reports on the simultaneous blockade of PD-1, other inhibitory receptors such as CTLA-4, Tim-3, Lag-3, and TIGIT have been shown to reactivate dysfunctional T cells and provide benefit from ICBs, which was also verified in our results (Figures 6F–I).

Therefore, the low-risk group was considered an immune “hot” phenotype, indicative of a beneficial response to immunotherapy. Alternatively, the high-risk group was identified as an immune “cold” phenotype, whose efficacy of immunotherapy was poor.

In our study, the constructed risk score consisted of only three pyroptosis-related lncRNAs, namely, AC090559.1, AC034102.8, and AC026355.2, which have been rarely mentioned in previous studies. Nevertheless, AC090559.1 was also identified as an independent risk factor related to autophagy and ferroptosis, suggesting that it may be a key regulator in programmed cell death (Guo et al., 2021; Wu et al., 2021). We also found that AC090559.1 may have a potential regulatory relationship with NLRC4. NLRC4 is the core component of NLRC4 inflammasomes, which was composed of a trigger (e.g., cytosolic flagellin), sensor (NAIP), nucleator (NLRC4), adapter (ASC), and effector (CASP1) (Duncan and Canna, 2018). The activation of NLRC4 inflammasomes can activate caspase-1, which cleaves pro-IL-1β and pro-IL-18 and simultaneously cleaves and activates gasdermin-D, thereby activating pyroptosis (Kay et al., 2020). Similar to pyroptosis, the effects of NLRC4 depend on the type and genomic background of the tumor. Continuously aberrant activation of chronic inflammation mediated by NLRC4 can promote the malignant progression of tumor cells. A previous study revealed that obesity-associated NLRC4 inflammasomes mediated IL-1β release, which promotes the growth of breast cancer by triggering VEGF production and angiogenesis (Kolb et al., 2016). Similarly, in nonalcoholic fatty liver, IL-1 signaling promoted metastasis (Ohashi et al., 2019). Consistent with our research, NLRC4 could also suppress tumor development while inducing antitumor immunity. In another study, Flagrp170, an artificially designed immunomodulator, showed protective antitumor immunity in an NLRC4-dependent manner (Yu X. et al., 2021). Sutterwala et al. demonstrated that NLRC4 enhanced inflammation in tumor-associated macrophages (TAMs) in a noninflammasome-dependent manner and the antimelanoma effects of IFN-γ produced by CD4 + and CD8 + T cells (Janowski et al., 2016). Moreover, NLRC4/NAIP5 also participated in the antigen recognition of flagellin-expressing tumor cells to facilitate antigen presentation to T cells, thereby activating CD4^+^ and CD8^+^ T cells and exerting antitumor effects (Garaude et al., 2012). However, studies on NLRC4 in lung cancer are still limited.

We found that NLRC4 was highly associated with immune cells such as activated/immature/plasmacytoid dendritic cells, γ delta T cells, MDSCs, macrophages, natural killer cells, regulatory T cells, T follicular helper cells, and type 1 T helper cells. Interestingly, AC090559.1 showed a similar effect compared with NLRC4 in the aforementioned immune cells (Supplementary Figure S3), suggesting that AC090559.1 may affect the extent of pyroptosis and the abundance of immune cells through NLRC4. Here, we performed correlation analysis of the three identified lncRNAs and several immune checkpoints and observed that these three lncRNAs were highly correlated with CTLA4 expression, suggesting that the risk model may reveal more significant effects on CTLA4 blockade; regardless of this, there were some limitations. Presently, there are few studies supporting the mechanisms of action of NLRC4 and AC090559.1, and thus, further studies are needed to clarify these mechanisms.

## Conclusion

In conclusion, we constructed a prognostic model based on pyroptosis-related lncRNAs. In addition to prognosis prediction, this model was significantly associated with pyroptosis extent and immune phenotype. We believe that these lncRNAs may serve as new targets for inducing pyroptosis, and stimulating pyroptosis-mediated antitumor immunity may provide new insights for the treatment of LUAD patients.

## Data Availability

The original contributions presented in the study are included in the article/Supplementary Material; further inquiries can be directed to the corresponding author.

## Supplementary Material

Supplementary Figure S1 Expression and prognosis analysis of NLRC4 and SCAF11/SIP1. **(A)** KM curves of NLRC4 and SCAF11/SIP1 in LUAD patients obtained from the KMPlot. Differential expression of NLRC4 and SCAF11 in GEPIA websites **(B)** and TIMER2.0 **(C)**. NLRC4 nod-like receptor family CARD domain containing 4, SCAF11/SIP1 SR-related CTD associated factor 11, LUAD lung adenocarcinoma.

Supplementary Figure S2 Differential abundance of infiltrating immune cells between high- and low-risk groups in LUAD. TME: tumor microenvironment, LUAD: lung adenocarcinoma.

Supplementary Figure S3 Correlation analysis between risk score, interacted mRNAs, identified lncRNAs, and TME infiltrating immune cells. TME: tumor microenvironment.

Supplementary Figure S4 Correlation between NLRC4 or SCAF11 and TME immune cell infiltration in LUAD from TIMER website. NLRC4: nod-like receptor family CARD domain containing 4, SCAF11: SR-related CTD associated factor 11, TME: tumor microenvironment, LUAD lung adenocarcinoma.

Supplementary Figure S5 Prediction of drug sensitivity.

## Glossary

AUC: Area under the curve
APC: Antigen-presenting cell
CCR: C-C chemokine receptor
ceRNA: Competing endogenous RNA
CNV: Copy number variations
CTL: Cytotoxic lymphocyte
CTLA-4: Cytotoxic T lymphocyte antigen-4
DAMP: Damaged-associated molecular pattern
FDR: False-discovery rate
FPKM: Fragments per kilobase of exon model per million reads mapped
GEPIA: Gene Expression Profiling Integrative Analysis
GSDMs: Gasdermins
HLA: Human leukocyte antigen
HR: Hazard ratio
ICB: Immune checkpoint blockade
ICP: Immune checkpoint
IFN: Interferon
IL: Interleukin
IPS: Immunophenoscore
KM: Kaplan–Meier
lncRNA: long noncoding RNA
LUAD: Lung adenocarcinoma
miRNA: MicroRNA
NK cell: Natural killer cell
OS: Overall survival
PCA: Principal component analysis
PCD: Programmed cell death
PD-1: Programmed cell death 1
ROC: Receiver operating characteristic
ssGSEA: Single-sample gene set enrichment analysis
TAM: Tumor-associated macrophage
TCGA: The Cancer Genome Atlas
TIDE: Tumor Immune Dysfunction and Exclusion
TME: Tumor microenvironment
