# Supplementary figures and images for: Identification and Validation of a Three Pyroptosis-Related lncRNA Signature for Prognosis Prediction in Lung Adenocarcinoma
Source: Front Genet. 2022 Jul 19;13:838624. doi: 10.3389/fgene.2022.838624 (PMC9345371; doi:10.3389/fgene.2022.838624)

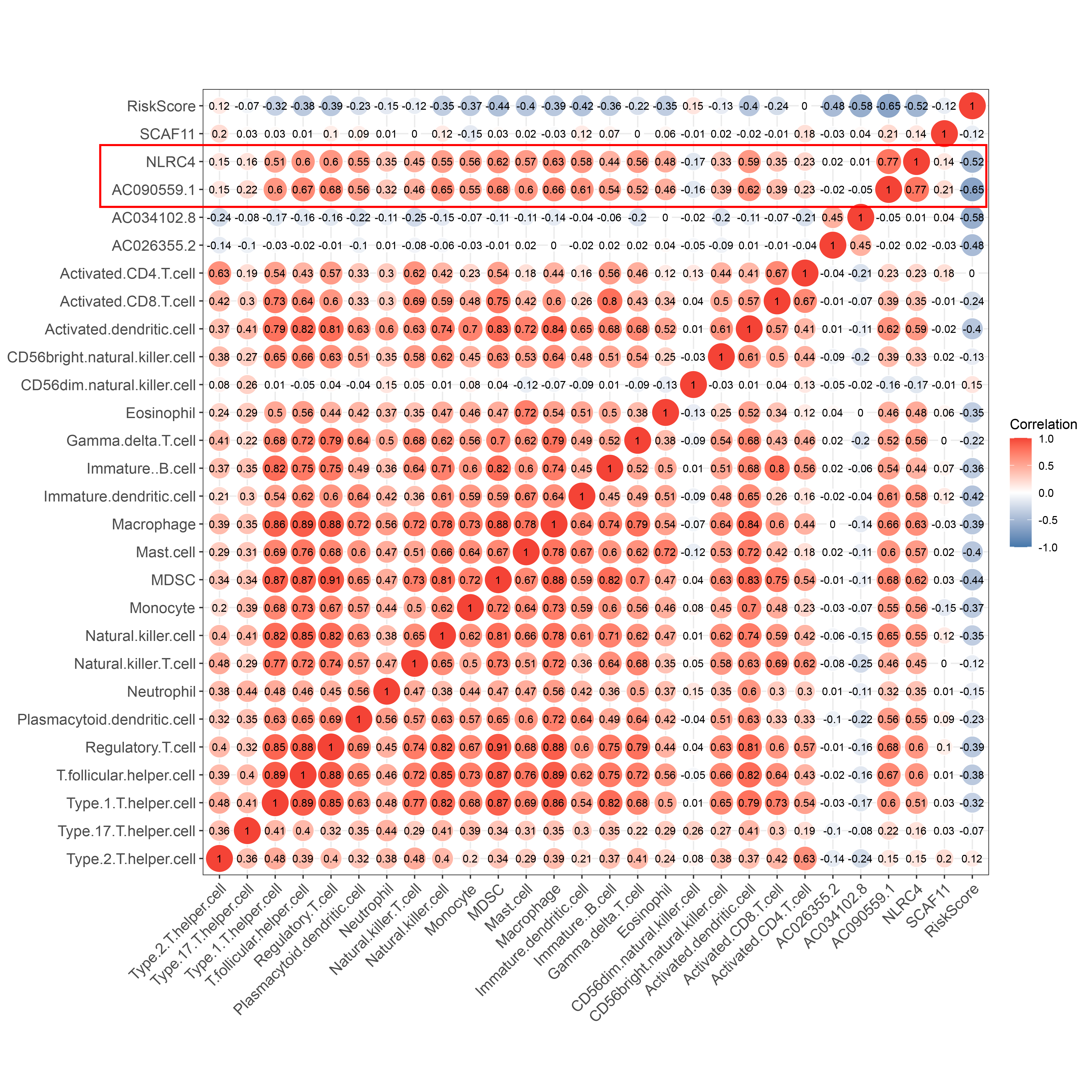

Supplement: Supplementary file 2 [file Image3.TIF]

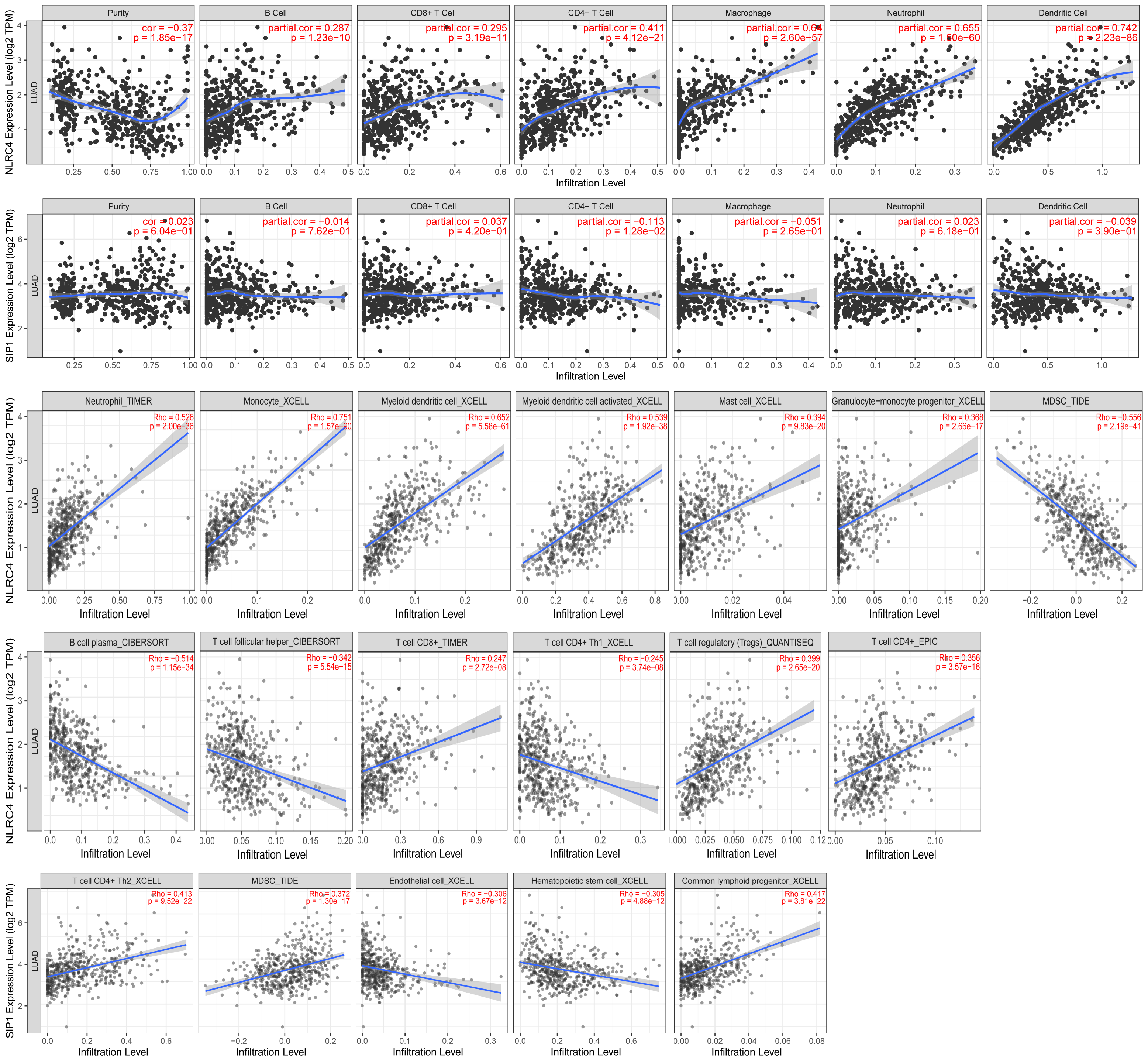

Supplement: Supplementary file 3 [file Image4.TIF]

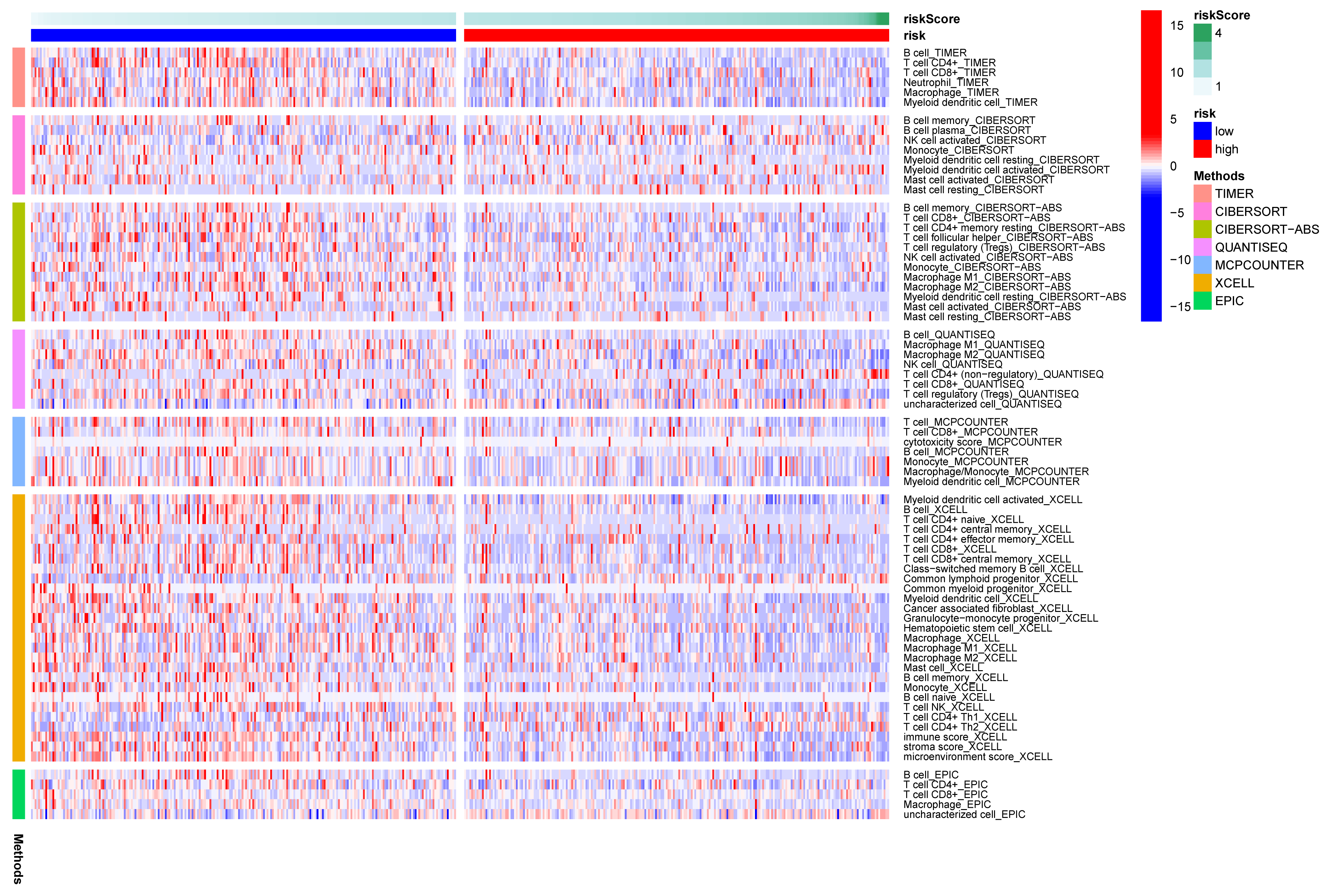

Supplement: Supplementary file 4 [file Image2.TIF]

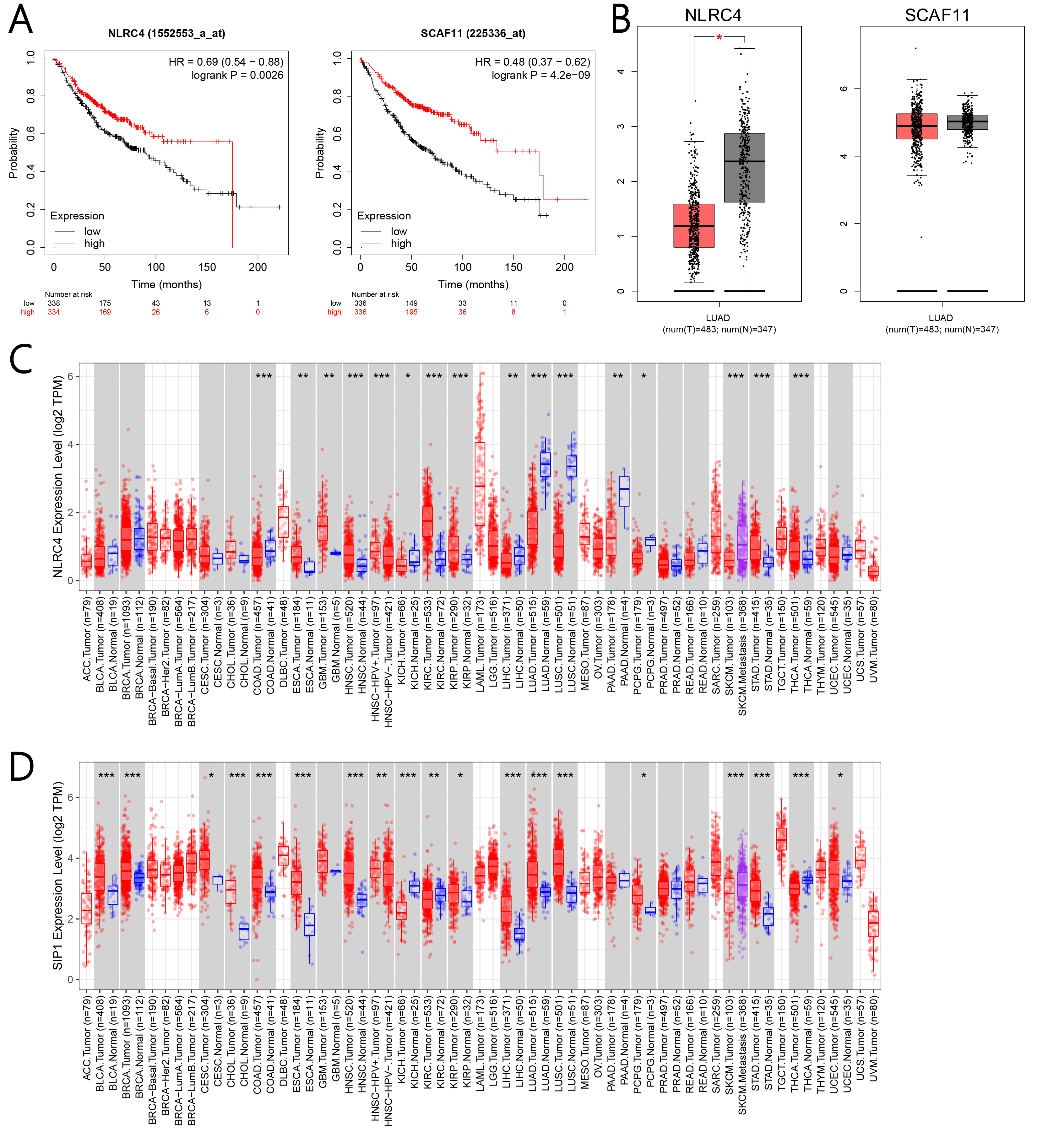

Supplement: Supplementary file 5 [file Image1.TIF]

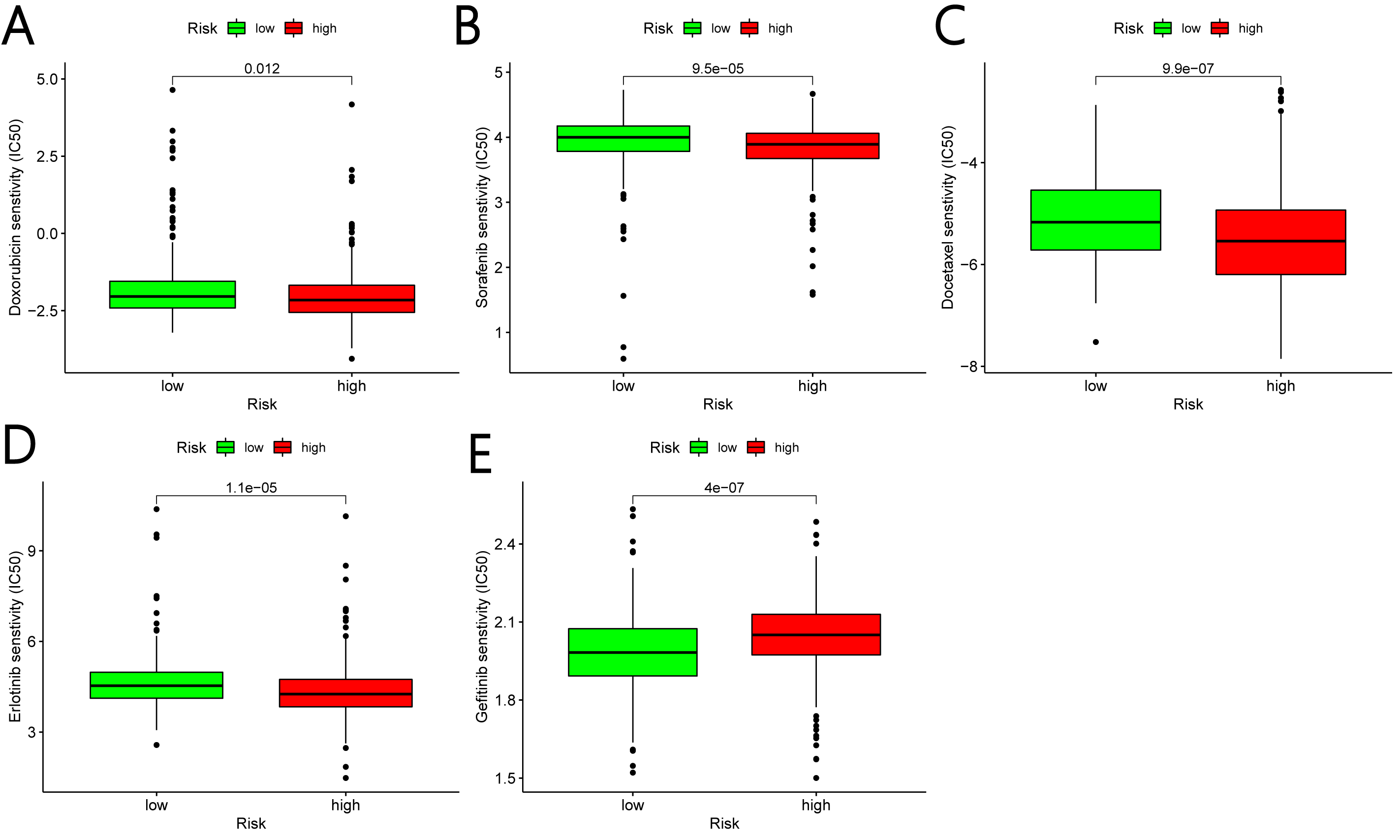

Supplement: Supplementary file 7 [file Image5.TIF]
