# Supplementary material for: Identification and Validation of a Three Pyroptosis-Related lncRNA Signature for Prognosis Prediction in Lung Adenocarcinoma
Source: Front Genet. 2022 Jul 19;13:838624. doi: 10.3389/fgene.2022.838624 (PMC9345371; doi:10.3389/fgene.2022.838624)
Supplement: Supplementary file 6 [file Table2.DOCX]

**Table S2.** **Primers used in this study**

| **gene** | **primer-F (5'-3')** | **primer-R (5'-3')** |
| --- | --- | --- |
| AC090559.1 | GTGAGTCAACCAAGCGAGAAGT | GGAAACTGAGGCAGGGACATC |
| AC026355.2 | CGTGGTAATCTGAGTGTGGTAAGC | GACCGATATGCCTATGCGAATGT |
| AC034102.8 | TGAGGACTACCCCAGCATAGG | CAGACAAAGGCATCTAAGAAAGGG |
| CD274 | GCTATGGTGGTGCCGACTACAA | GGTGGTGGTCTTACCACTCAGGA |
| PDCD1 | AAACCCTGGTGGTTGGTGTC | TGGCTCCTATTGTCCCTCGT |
| CTLA4 | GGATTTCAGCGGCACAAGG | CCTGGAGATGCATACTCACACACA |
